# DICER1 Syndrome in Twins With Ovarian Sertoli-Leydig Cell Tumor and Papillary Thyroid Carcinoma

**DOI:** 10.7759/cureus.41344

**Published:** 2023-07-04

**Authors:** Hessah A Al Hussaini, Maswood M Ahmad, Majed Albarrak, Suphia M Sherbeeni, Omar AlHuzaim

**Affiliations:** 1 Internal Medicine, King Faisal University, Al Ahsa, SAU; 2 Diabetes and Endocrinology, Obesity and Endocrine Metabolism Center, King Fahad Medical City, Riyadh, SAU; 3 Otolaryngology, King Fahad Medical City, Riyadh, SAU; 4 Endocrinology, Diabetes and Metabolism, Nera Medical Specialist and Day Surgery Center, Riyadh, SAU; 5 Adult Endocrinology, Obesity and Endocrine Metabolism Center, King Fahad Medical City, Riyadh, SAU

**Keywords:** dicer1 syndrome, thyroidectomy, ovarian sertoli leydig cell tumors, papillary thyroid carcinoma, multinodular goiter, twins

## Abstract

DICER1 syndrome is a rare autosomal dominant syndrome resulting in benign and malignant tumors in various organs with tumors in endocrine organs (pituitary, thyroid, adrenal, ovaries, and pancreas). Here we present a rare case of 18-year-old twin sisters with DICER1 syndrome who presented at the age of 15 years with hirsutism, deepening of the voice, and amenorrhea. They were diagnosed with a Sertoli-Leydig cell tumor of the ovary and underwent unilateral oophorectomy, with no evidence of recurrence or metastasis during follow-up. Genetic analysis showed the same germline DICER1 mutation in both cases. They also had large multinodular goiters (nodule size ranging from 1.0 to 2.3 cm) nodules were increasing in size. Fine needle aspiration cytology (FNAC) of thyroid nodules for both the sisters showed atypia of undetermined significance/follicular lesion of undetermined significance (AUS/FLUS), and they both underwent total thyroidectomy revealing papillary thyroid carcinoma. No pituitary lesion was observed in the brain magnetic resonance imaging (MRI) of either of them. A chest CT scan showed bilateral sub-pleural benign-looking nodules in both patients. The twin sisters developed some features, such as Sertoli-Leydig cell tumor, multinodular goiter, and papillary thyroid carcinoma, and had positive genetic tests for DICER1 germline mutation. The father and paternal grandfather had a family history of papillary thyroid carcinoma. Both patients require active surveillance due to the increased risk of developing tumors in multiple organs associated with this disease.

## Introduction

DICER1 syndrome is a rare autosomal dominant, pleiotropic tumor predisposition disorder [1]. It is caused by pathogenic germline variation in the DICER1 gene with or without concurrent somatic mutation (Knudson’s two-hit hypothesis). DICER1 gene is located on chromosome 14 q32.13, and is a member of the RNAse III family with an essential role in the formation of mature microRNA [2], and the tumorigenesis is promoted by the downregulation of microRNA formation [3]. DICER1 syndrome results in a variety of tumors which can be both benign and malignant [1]. Most of them develop before 40 years of age [4]. Certain tumors are considered the hallmark of the disease, such as pleuropulmonary blastoma, Sertoli-Leydig cell tumor of the ovary, and cystic nephroma, and the presence of these tumors signals the testing for DICER1 gene mutation [1-4]. Apart from these tumors, DICER1 syndrome is associated with a 13-75% risk of developing thyroid nodules/cancer [4-6]. Indications for genetic testing and counseling in patients suspected of DICER1 syndrome and surveillance strategies in those with positive germline DICER1 mutation have been suggested to guide and manage such patients appropriately, according to which genetic testing should, ideally, be performed on a proband with a DICER1 associated tumor when available. Major and minor indications for genetic testing have been suggested, and genetic testing and counseling are also recommended for patients with a history of at least one major or two minor indications. In the absence of major indications in an individual, the presence of one minor indication and a family history of a major or minor indication are considered sufficient enough for genetic testing [1]. International Pleuropulmonary Blastoma (PPB) Registry and International Ovarian and Testicular Stromal Tumor (OTST) Registry are the official registries that have been identifying all cases of DICER1 syndrome-related tumors [1], in addition to a few published case reports about siblings with same syndrome [3-7]. No previous report about twin siblings with DICER1 syndrome has been reported so far. Herein, we present a rare case of twin sisters with DICER1 syndrome involving two different endocrine organs.

## Case presentation

Case 1

**Figure 1:**
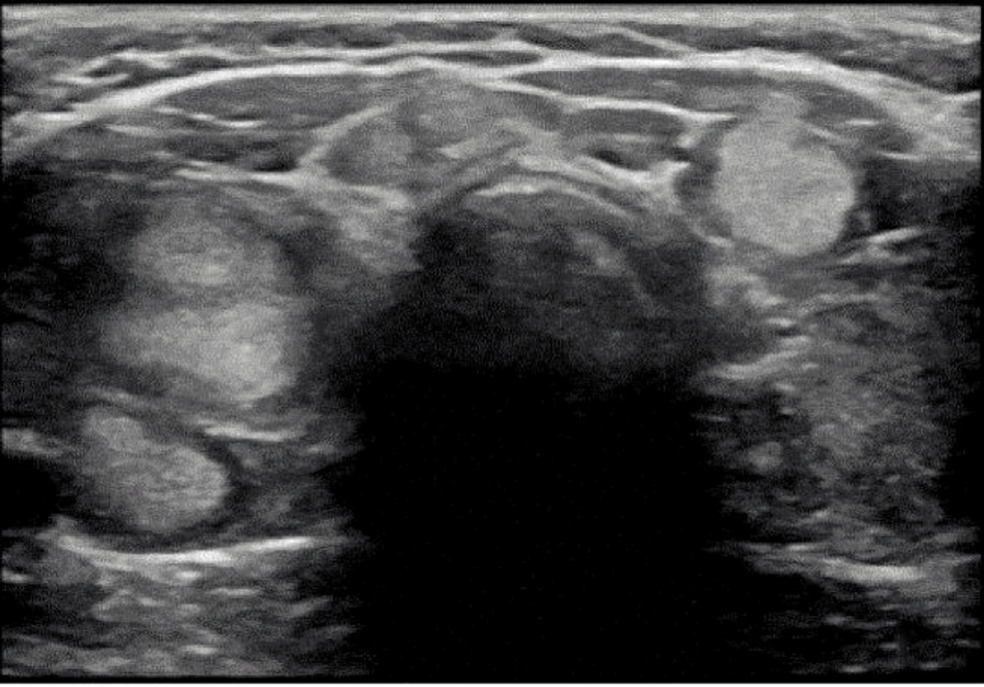
Thyroid ultrasound of Case 1 showing multinodular goiter with nodules size ranging from 1 to 2.3 cm.

Case 1 was an 18-year-old girl with primary hypothyroidism receiving levothyroxine replacement therapy. At the age of 15 years, she presented with hirsutism, deepening of the voice, and amenorrhea. She was diagnosed with a Sertoli-Leydig cell tumor of the ovary and underwent unilateral oophorectomy. Her eyes and teeth were appearing normal on examination and ultrasonography of the urinary system showed no structural anomalies. She remained in remission during follow-up until now and had no clinical, biochemical, or radiological evidence of recurrence or metastasis. Genetic analysis was performed which revealed a germline DICER1 mutation. Given the positive finding of DICER1 mutation, she underwent screening for tumors in other organs, and a large multinodular goiter was found with nodule sizes ranging from 1 to 2.3 cm (Figure 1).

The father and paternal grandfather had a family history of papillary thyroid carcinoma. Their father did not carry the same genetic mutation while the mother had no history of any malignancies, goiter, or thyroid dysfunction. The genetic status of the mother was unknown for the DICER1 mutation as she refused to be screened. The thyroid nodules were increasing in size, and she underwent fine needle aspiration cytology (FNAC) from nodule which showed atypia of undetermined significance/follicular lesion of undetermined significance (AUS/FLUS) cytology. Total thyroidectomy was performed which showed histopathological features diagnostic for low-risk papillary thyroid carcinoma with a tumor size of 0.2 cm (T1N0Mx) that did not require adjuvant therapy. The patient’s blood work for other hormonal assays was found to be within normal limits (Table 1, Case 1).

**Table 1:** Laboratory workup of Case 1 and Case 2. CA: cancer antigen; CEA: carcinoembryonic antigen; DHEA-S: dehydroepiandrosterone sulfate; ACTH: adrenocorticotropic hormone; LH: leutenizing hormone; FSH: follicle-stimulating hormone; TSH: thyroid-stimulating hormone; IGF-1: insulin-like growth factor 1

Variables	Case 1	Case 2	Normal values
CA 125	11.6	16.3	0.00-35 KU/L
CA 15-3	6.8	8.7	0.00-25 KU/L
CA 19-9	14.2	14.6	0.00-27 KU/L
CEA	0.9	0.9	0.00-4.7 ng/ml
DHEA-S	2.72	1.83	0.62-4.93 µg/mL
ACTH	16.34	9.53	7.26-63.11 pg/mL
Short Synacthen test-peak serum cortisol	27.4	30.19	3.66-19.43 µg/dL
LH	6.1	2.5	Women, follicular phase of menstrual cycle: 1.68 to 15 IU/L women, midcycle peak: 21.9 to 56.6 IU/L women, luteal phase: 0.61 to 16.3 IU/L
FSH	4.52	2	Premenopausal women: 4.7 to 21.5 IU/L
Testosterone total	0.424	0.412	0.1-0.56 ng/mL
Free testosterone index	4.4%	5.4%	Not available
Sex-hormone binding globulin	3.44	2.51	1.08-13.01 µg/mL
Estradiol	77.62	100.5	Premenopausal women: 30 to 400 pg/mL
TSH	2.87 (on thyroid replacement)	1.11	0.40-4.50 mIU/mL
IGF-1	Not done	361.79	161.39-580.55 ng/mL
Prolactin	5.1	8.93	Nonpregnant women: 2 to 29 ng/mL

**Figure 2:**
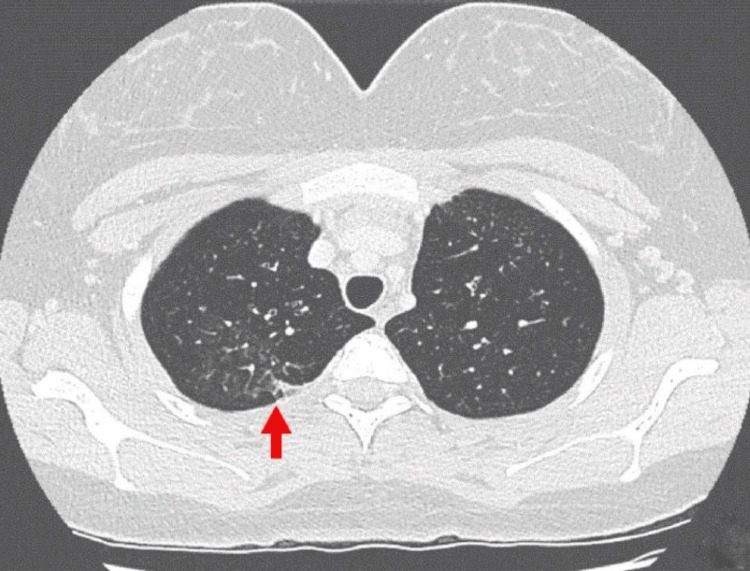
Chest computed tomography of Case 1 showing benign-looking subpleural nodules (arrow).

Her pituitary MRI did not reveal any abnormality. Currently, she is under active surveillance with thyroid examination, thyroid tumor markers, and thyroid ultrasound. CT scan of the chest was performed as part of surveillance where she was found to have bilateral sub-pleural benign-looking nodules (Figure 2).

Case 2

**Figure 3:**
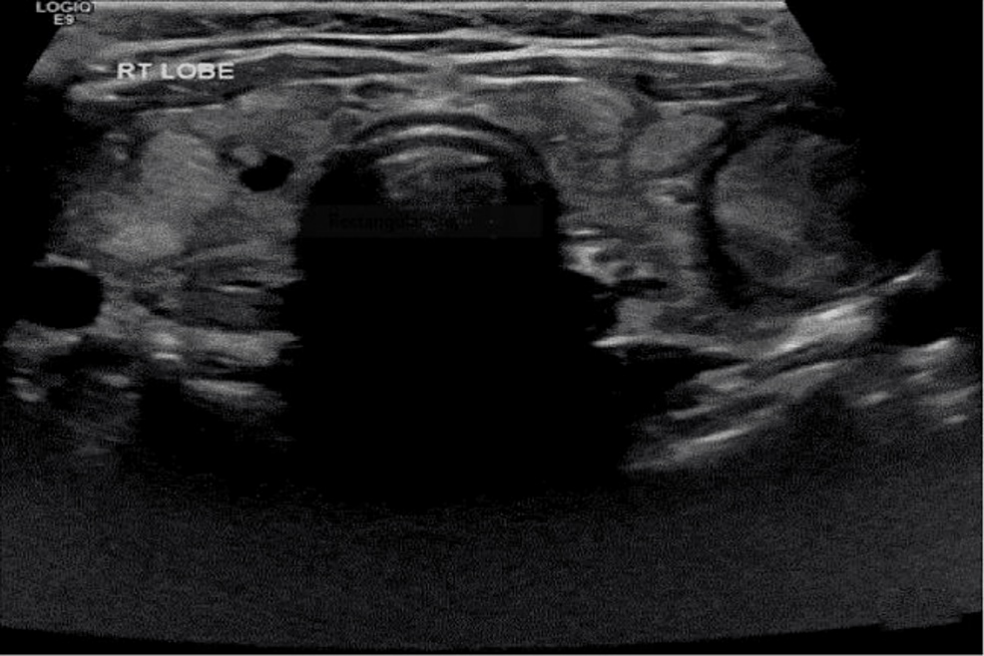
Thyroid ultrasound of Case 2 showing multinodular goiter with varying sizes of nodules.

Case 2 was an 18 years old girl and the twin sister of Case 1. She had a similar presentation to her sister, and at the age of 15 years, she also presented with hirsutism, deepening of the voice, and amenorrhea. She was diagnosed with a Sertoli-Leydig cell tumor of the ovary and underwent unilateral oophorectomy, again without any evidence of recurrence or metastasis during follow-up. Her physical examination was normal with no abnormalities in her eyes or teeth and no structural anomaly was observed in ultrasonography of the urinary system. She also had the same germline DICER1 mutation as her sister. Upon surveillance, she was also found to have a large multinodular goiter with increasing and varying sizes of nodules (Figure 3).

**Figure 4:**
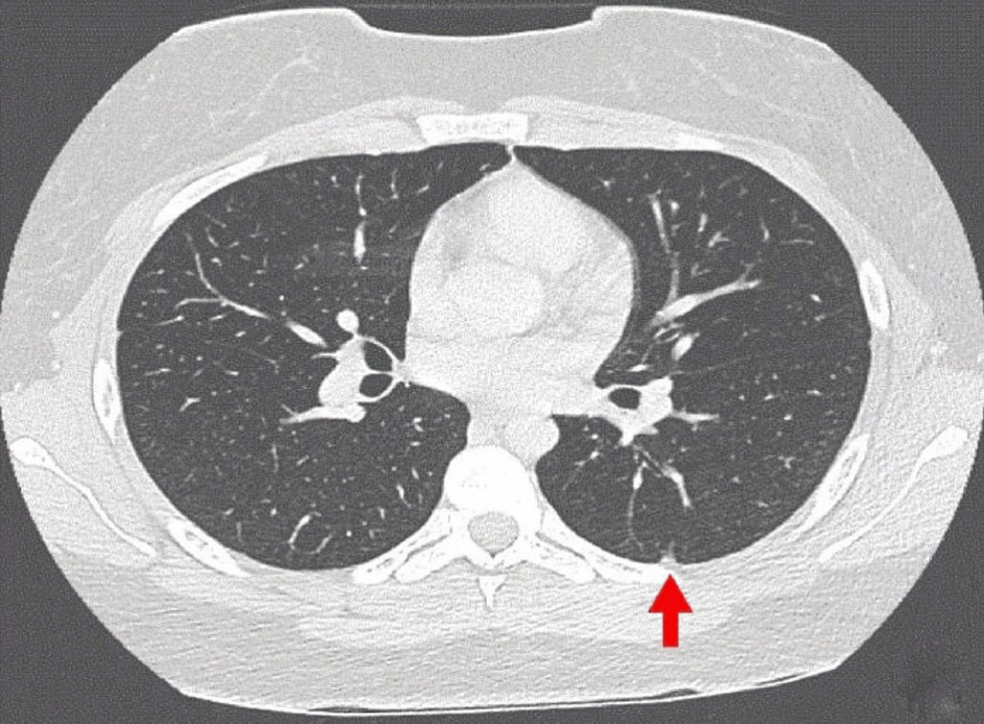
Chest computed tomography of Case 2 showing benign-looking subpleural nodules (arrow).

In contrast to her twin sister, she had normal thyroid function and did not require any hormonal replacement. Her FNAC findings were similar to those of her twin sister as AUS/FLUS. Subsequently, she also underwent total thyroidectomy which revealed papillary thyroid carcinoma with follicular differentiation, a tumor size of 3.0 cm, and no capsular invasion. Her pituitary hormonal profile was within normal limits without any pituitary lesions on the pituitary MRI (Table 1, Case 2). The patient was also screened for pleuropulmonary blastoma by chest CT, which showed bilateral sub-pleural benign-looking nodules (Figure 4).

## Discussion

DICER1 syndrome is a rare autosomal-dominant pleiotropic tumor-predisposition disorder resulting in an increased risk of tumors in various organs which can be both benign as well as malignant [1]. Predisposition for tumor formation is mainly observed in the lungs, ovaries, thyroid, and cystic nephroma. Pituitary blastoma, pineoblastoma, CNS sarcoma and other tumors, ciliary body medulloepithelioma, embryonal rhabdomyosarcoma, nasal chondromesenchymal hamartoma, and pre-sacral malignant teratoid tumor are some other less commonly observed malignancies. Structural anomalies of the kidney and collecting system, ocular abnormalities, macrocephaly, and dental anomalies may also be present [4]. 

DICER1 belongs to the ribonuclease III (RNase III) family of genes which are involved in the processing of pre-microRNA into mature active microRNAs. These short, double-stranded microRNAs are non-coding regulatory RNAs having important roles in metabolism, cell proliferation, morphogenesis, apoptosis, and modulating gene expression post-transcriptionally [8]. Global downregulation of these microRNAs is associated with tumor predisposition [9].

The DICER1 is a haploinsufficient tumor suppressor gene located on chromosome 14 q32.13. Pathogenic germline mutations with possible concurrent somatic mutation of the DICER1 gene result in DICER1 syndrome [9,10]. In DICER1-associated tumors, the loss of function variant is seen in the germline copy while the wild-type copy is disrupted by a somatic hotspot variant [11]. Somatic variations have been observed in DICER1-associated tumors at amino acid “hotspot” residues located in the metal binding domain of RNAse IIIb leading to disruption of enzymatic function [12].

Approximately 80% of DICER1 gene mutations are inherited, whereas 20% are hypothesized to occur de novo [4]. They exhibit low penetrance in pleuropulmonary blastoma and cystic nephroma but show high penetrance in multinodular goiter [10]. The estimated prevalence of individuals carrying the pathogenic DICER1 gene variant is 1:2,529 to 1:10,600 in the general population [1,13], and 1:4,600 in adult and pediatric cancer populations in The Cancer Genome Atlas (TCGA) [2]. For patients harboring DICER1 germline gene mutation, the risk of developing tumors is highest among children. However, the incidence decreases during adulthood, as most of the tumors usually develop before 40 years of age and little is known about the risk of malignancies or other conditions in older patients [4,14].

From 1996 to 2011, the International Pleuropulmonary Blastoma (PPB) registry and International Ovarian and Testicular Stromal Tumor (OTST) registry have identified 500 and 160 cases of sex cord-stromal tumors associated with DICER1 gene mutation, respectively [1]. Following an initial diagnosis of DICER1 tumor predisposition in an individual, the two things that need to be considered are the staging or extent of disease spread for malignant or potentially malignant tumors and active surveillance for other DICER1-associated conditions [4]. Among associated multisystem tumors, endocrine tumors have been linked to DICER1 syndrome, specifically pituitary blastoma (most commonly presenting in young patients with Cushing disease), pancreatic intraepithelial neoplasms, adrenocortical adenoma and carcinoma along with thyroid nodular diseases and cancers [5].

In addition to differentiated thyroid cancer, the risk of multinodular goiter is increased in patients with DICER1 syndrome compared to population data found in the National Cancer Institute and Surveillance, Epidemiology and End Results program [2]. In patients with DICER1 gene mutations, multinodular goiter develops in 32% of women and 13% of men by 20 years of age and 75% of women and 17% of men by 40 years of age, with or without the need for thyroidectomy [4,6,15], with 20% higher risk for thyroid cancer [5]. Compared to the general population, the risk of differentiated thyroid cancers (papillary thyroid carcinoma, follicular variant of papillary thyroid carcinoma, and follicular thyroid carcinoma) is 16-24 times higher in these patients [1-4].

The higher risk of differentiated thyroid cancer was thought to be secondary to an increased prevalence of thyroid nodules and chemotherapy and radiotherapy received as treatment for other malignancies in patients with DICER1 syndrome. However, even patients without these risk factors for differentiated thyroid cancer can harbor somatic mutations of the DICER1 gene [6]. DICER1 syndrome-associated differentiated thyroid cancer usually has indolent behavior with favorable outcomes and is often encapsulated [4]. Lymphovascular invasion, extrathyroidal extension, or metastasis to regional lymph nodes is uncommon [16]. Interestingly, familial multinodular goiter secondary to DICER1 gene mutations can present as multinodular goiter and Sertoli-Leydig cell tumors without the coexistence of pleuropulmonary blastoma [3,5,7].

For the rare Sertoli-Leydig cell tumor, up to 60% of cases carry a pathogenic DICER1 gene variant (with or without the presence of multinodular goiter) [13,15]. Though it can occur at any age, it is seen most commonly in adolescents and young adults and usually presents with abdominal pain, abdominal mass, and distension. Amenorrhea, menstrual irregularity, precocious puberty, and features of virilization can also be seen. Gynandroblastoma is another sex cord-stromal tumor of the ovary seen in girls and young women and can present with or without signs of excess hormonal secretion [4].

Although being a rare entity, indications of genetic testing and counseling have been well outlined and major and minor indications have been suggested as per current understanding of the disease pathology and phenotypes. Apart from a proband presenting with DICER1-associated malignancies, genetic testing and counseling are recommended for patients with a personal history of at least one major or two minor indications when available. The presence of one minor indication along with a family history of a major or minor indication is also considered sufficient enough for genetic testing in the absence of major indications [1]. Findings such as any type of pleuropulmonary blastoma, bilateral/multiple or multiseptated pulmonary cysts in children, embryonal rhabdomyosarcoma of thorax, ovary, cervix, and uterus, genitourinary sarcoma, cystic nephroma, ovarian Sertoli-Leydig cell tumor, gynandroblastoma, gynecological or genitourinary neuroendocrine tumors, multinodular goiter or differentiated thyroid carcinomas of childhood-onset, or occurring in two or more first-degree relatives or in an index case with a positive family history consistent with DICER1 syndrome, nasal chondromesenchymal hamartoma, ciliary body medulloepithelioma, pineoblastoma, and pituitary blastoma have been considered as major indications for genetic testing. Minor indications of testing are pulmonary cysts in adults, Wilms tumor and renal cysts, multinodular goiter and differentiated thyroid carcinomas, poorly differentiated neuroendocrine tumors, embryonal rhabdomyosarcomas of non-thoracic and non-gynecological origin, undifferentiated sarcoma and macrocephaly. Testing is also to be considered for any childhood malignancy in constellation with any other minor indications [1].

Recommendations for screening and surveillance have been suggested similarly with a goal to detect tumors at the earliest stage when they are more amenable to curative therapy and thus prolong disease-free survival in them. For pleuropulmonary blastoma, all children at risk of pathogenic germline DICER1 mutation should be screened with chest X-ray at birth to detect large pulmonary cysts. Molecular testing for DICER1 mutation should be obtained before three months of age and those found to be carriers of pathogenic germline DICER1 mutation should get their first chest CT scan done preferably at three to six months, however, before nine months of age. Ultrasonography of abdomen can be done at the same time with CT chest as screening for renal involvement. For female reproductive tract involvement, health education for patients and family members regarding clinic features, such as abdominal pain, abdominal lump, or hormonal symptoms, are of utmost importance as pelvic ultrasonography is required urgently in case of appearance of such manifestations. Pelvic ultrasonography can be obtained together with abdominal ultrasonography during infancy and then every six to 12 months [1]. Patients and their family members need to be educated about increased risk of thyroid involvement in the form of goiter, nodules, and carcinomas. Despite the absence of any prospective studies regarding the timing or efficacy of thyroid ultrasonography in patients harboring pathogenic germline DICER1 mutation. Thyroid ultrasound is recommended by the age of eight years and every three to five years as surveillance for thyroid nodules. It can be done earlier if the patient has been exposed to chemotherapy or radiotherapy for non-thyroid cancers. In such scenarios, a baseline thyroid ultrasonography is indicated at the time of diagnosis and then annually for five years after exposure. FNAC should be obtained from all thyroid nodules and thyroidectomy is considered for symptomatic nodules, nodules showing significant growth on serial ultrasonography, or those with abnormal cytology. A thorough assessment of lateral neck lymph nodes by ultrasonography is a must before thyroidectomy to include lateral compartment neck dissection at the time of first surgery if needed [1,4].

Through early detection of tumors by imaging of multiple organs, these proposed surveillance protocols and strategies target to reduce the morbidity and mortality associated with DICER1 syndrome. However, in a recent article, Bakhuizen et al. discussed some drawbacks and questioned the clinical utility of these protocols as they are yet to be validated. The growth rate and natural history of most DICER1-associated tumors are not known with certainty and have not been investigated. Overtreatment in the form of unnecessary surgery for asymptomatic benign cysts, radiation exposure, imaging procedures requiring sedation in young children, psychosocial burden of repeated investigations, and consequences of false-positive findings are some of the potential harms associated with these protocols [17].

Our patients had germline mutations of the DICER1 gene and some of the features of the syndrome, such as Sertoli-Leydig cell tumor of the ovary, multinodular goiter, and differentiated thyroid cancer. They had no dental and ocular abnormalities on physical examination, and the renal and collecting systems revealed no abnormality on ultrasonography. Both patients had enlargement of goiter with increasing size of the nodules and showed AUS/FLUS cytology on FNAC. Total thyroidectomy was required where papillary carcinoma of the thyroid was found in either of them. Both patients had no previous history of chemotherapy or radiation exposure. They had a strong family history of papillary thyroid carcinoma in their father and grandfather [1,6]. Although a rare disease, it is associated with significant morbidity risk, and it is crucial to understand the importance of identifying patients with DICER1 gene mutation and their need for active surveillance for underlying tumor development [13].

## Conclusions

The overall prognosis in patients with DICER1 mutation is good as most of the individuals are otherwise healthy or have only one DICER1-associated condition. Early diagnosis and appropriate surveillance strategies are helpful in achieving prolonged disease-free survival and cure. There are various research questions that are yet to be answered, such as the growth rate and natural history of DICER1-associated tumors, epidemiology of tumors in adults, and the clinical utility of the current surveillance protocols. At the same time, more research is required to increase our knowledge of the phenotypes of the DICER1 gene mutation, including thyroid and pituitary malignancies, especially for patients who did not receive chemotherapy or radiotherapy like our patients. With increase in our knowledge about the DICER1 syndrome and its clinical implications, revision of criteria for genetic testing and optimization of surveillance protocols are going to decrease the associated healthcare expenditure. Education and counseling of patients and their family members is of utmost importance. Our patients need active surveillance for their potential risk of developing multiorgan tumors associated with DICER1 gene mutation.
